# Troublesome Tuberculosis: A Case Report on Multi-focal Tuberculous Osteomyelitis in An Immunocompetent Patient

**DOI:** 10.4021/jocmr758e

**Published:** 2012-01-17

**Authors:** Myo M. Lynn, Jeeva R. Kukanesen, Abdul W. Khan

**Affiliations:** a Rheumatology Department, Queen Elisabeth Hospital, London, UK; b Geriatric Medicine Department, King’s College Hospital, London, UK; c Rheumatology Department, St George’s Hospital, London, UK

## Abstract

Bone and joint tuberculosis is a chronic debilitating condition that leads to progressive damage and even deformity of joints. It may affect one or multiple sites. It could present in a myriad of ways which may result in an incorrect diagnosis being made. Common misdiagnoses include seronegative inflammatory arthritis, septic arthritis, malignancy, osteoporotic fractures and mechanical type back pain. It was initially only diagnosed in patients with previous active tuberculosis or latent tuberculosis. However, in recent years, it has also been reported in patients without a history of previous tuberculosis infection. Making a diagnosis of bone and joint tuberculosis is challenging. As the symptoms are not always typical, a delay in initiating anti-tuberculosis treatment is not uncommon in clinical practice. Systemic features are not always present in multi-drug resistant tuberculosis of joints which makes the diagnosis even more challenging. Multi-drug resistant tuberculosis is an increasingly common problem. It is not only limited to immunocompromised patients, but also found in immunocompetent patients. Multifocal tuberculous osteomyelitis is an uncommon condition and may involve any bone such as the skull, ribs, long bones, spine and phalanx. Tuberculous pyomyositis and tuberculous tenosynovitis may also be the presenting features of multifocal tuberculosis. Identification of mycobacterium tuberculosis in synovial fluid and biopsy, tissue culture, tissue fluid cytology and tissue polymerase chain reaction are crucial investigations in these cases. As the presentation of extra pulmonary tuberculosis can be very variable, it is important to maintain a high index of suspicion. The diagnosis and therefore treatment may be expedited using a clinically directed multidisciplinary approach.

**Keywords:**

Bone and joint tuberculosis; Multi focal tuberculous osteomyelitis; Extra-pulmonary tuberculosis; Multi-drug resistant tuberculosis; Latent tuberculosis

## Introduction

Bone and joint tuberculosis (TB) is one of the many extra-pulmonary manifestations of tuberculosis. It classically affects the weight bearing joints such as the knees and hips. It is usually, but not always, monoarticular in nature. Multifocal tuberculous osteomyelitis is an uncommon condition and may involve any bone such as the skull, ribs, long bones, spine and phalanx. Tuberculous pyomyositis may also be a presenting feature of multifocal tuberculosis. Tuberculous tenosynovitis could be mistaken as a chronic tenosynovitis. Sternal tuberculosis may present as long standing chest pain [1] which could potentially lead to a very different clinical diagnosis. Back pain due to spinal tuberculosis usually results in a wide range of differential diagnosis. There has also been a case report of spinal TB and paravertebral abscess following a kyphoplasty [2].

Among the various forms of extra pulmonary tuberculosis, tuberculous dactylitis and polyarticular tuberculosis are recognised presentations in patients who live in countries with a high prevalence rate of tuberculosis. It is frequently associated with disseminated disease [3]. Typical sinus formation of dactylitic bone may not always be present. Concurrent pulmonary tuberculosis and systemic features such as fever, weight loss and night sweats may or may not be present in bone and joint tuberculosis. This therefore is a condition which could be mistaken as a seronegative inflammatory arthritis and hence delay initiating appropriate therapy.

We report on a case of an immunocompetent fifty two year old man who presented with multifocal polyarticular multi-drug resistant tuberculosis. He was initially diagnosed as a seronegative inflammatory arthritis and subsequently as a possible septic arthritis. He failed to respond to a trial of intravenous antibiotics. A definitive diagnosis was finally achieved with the detection of mycobacterium tuberculosis from the synovial fluid as well as granulomatous changes seen in a synovial tissue biopsy.

## Case Report

Fifty two year old man of Chinese descent was admitted to hospital with epistaxis and bleeding per rectum. This was a consequence of a high international normalized ratio (INR) due to over-anticoagulation with warfarin. The language barrier impaired our ability to obtain a detailed history. It was incidentally noted that he had a swollen left knee and leg for about three months. It was initially thought to be a deep vein thrombosis and was treated with anti-coagulant, warfarin. A knee aspiration showed haemarthrosis which then required an ultrasound guided joint wash out. The knee aspirate was sent for microscopy, culture and sensitivity and initial results came back negative. The patient was discharged home with conservative treatment. Two week later, he represented to hospital with fever and worsening knee pain. Further detailed history through a trained translator brought to light that he had in fact been having recurrent asymmetrical polyarthritis for almost five years prior to migrating to the United Kingdom.

His blood results showed a mild anaemia with a haemoglobin of 10.0 G%, normal white cell count, normal neutrophil count and high inflammatory markers with a C-reactive protein (CRP) of 44. His erythrocyte sedimentation rate (ESR) was 62. He had a normal chest X-ray and normal urinalysis. His rheumatoid factor and anti-cyclic citrullinated protein antibody (anti-CCP) were negative. His HIV test which was performed at an early stage came back negative.

**Figure 1:**
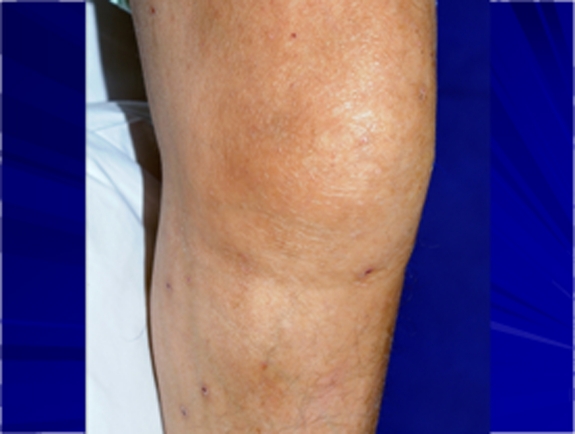
Tuberculous septic arthritis in left knee.

**Figure 2:**
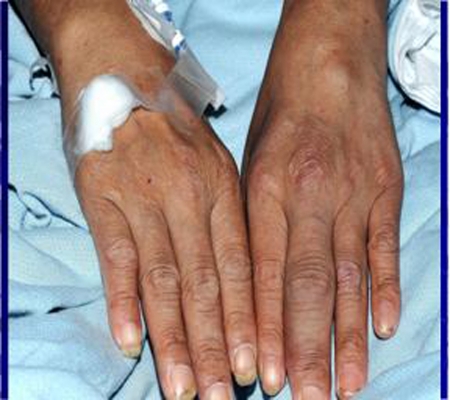
Tuberculous dactylitis in left middle finger.

Clinical examination revealed swollen and tender joints in the right elbow, right wrist, left knee (Fig. 1) and left ankle which were consistent with active synovitis. He also had dactylitis (Fig. 2) in the left middle proximal interphalangeal joint. He was mildly pyrexial with a temperature of 37.8 degrees Celsius. His inflammatory markers were persistently high during his admission. Knee joint aspiration was repeated and samples were sent again for microscopy, culture and sensitivity for bacteria, viruses and mycobacterium tuberculosis.

A differential diagnosis of polyarticular septic arthritis had been considered and intravenous flucloxacillin two grams every six hours was initiated on the advice of the Microbiologist. The fever settled initially and there was a minimal improvement in his inflammatory markers. The intravenous antibiotic was continued for two weeks but there was no further improvement in his inflammatory markers and his fever returned. A concomitant tuberculosis infection was still considered a possibility despite the earlier negative results. A Mantoux test performed was also normal.

As the patient continued to have fevers and raised inflammatory markers despite the antibiotics, the possible alternative diagnosis of seronegative inflammatory arthritis was re-considered. Further investigations including a computerised tomographic (CT) scan of his chest and abdomen did not show any obvious pathology apart from small volume hilar lymphadenopathy. Magnetic resonance imaging (MRI) of his pelvis and sacroiliac joints showed no evidence of sacroilitis or a psoas abscess. In view of his mild anaemia, an upper gastrointestinal endoscopy was done which revealed a duodenal ulcer with positive helicobacter pylori. He was treated with eradication antibiotics therapy. As a series of blood cultures and urine cultures were negative, and there was no obvious clinical improvement, the antibiotics were stopped. The Orthopaedic team performed a synovial biopsy from his knee. The result showed granulomatous changes and the synovial tissue was sent for polymerase chain reaction (PCR). Sarcoidosis and tuberculosis were suggested as possible differential diagnosis.

After eight weeks in hospital, mycobacterium tuberculosis was grown from the initial synovial fluid sample taken from the knee. An isotope bone scan was performed and it has shown an increased uptake at the wrist, ribs, knees and ankles consistent with inflammatory changes. A diagnosis of multi-focal tuberculous osteomyelitis was made and standard quadruple therapy was initiated. Over the next two weeks, the fever settled and his acute phase markers also gradually improved. He was discharged after two weeks of initiating the anti-tuberculous treatment with an out-patient follow-up appointment.

One month after discharge, full sensitivity result from synovial fluid culture was available and it has shown that the patient was found to be resistant to Rifampicin, Isoniazid, and Ethambutol, but sensitive to Pyrazinamide, Amikacin, Moxifloxicin, Protionamide and Cycloserine. Given the complexity of treating multi-drug resistant tuberculosis, he was readmitted to a tertiary hospital where experts with experience in this area are available.

In this case series, the time from presentation to hospital to getting a correct diagnosis, was approximately eight weeks.

## Discussion

Tuberculosis is still a leading cause of death among the infectious diseases. Because of current migratory patterns, tuberculosis has become a global concern. The World Health Organisation declared tuberculosis a global emergency in 1993. According to the Health Protection Agency UK (HPA), provisional data has shown that 8587 cases of tuberculosis were reported in the UK in 2010. This is a rate of 13.9 per 100 000 population. This is a 6% decrease data compared to provisional number reported in 2009 [4]. In 2008, 334 patients died from tuberculosis which is 0.6 per 100 000 population. This data is significantly better compared to 1955 in which 3900 patients died from tuberculosis with mortality rate of 8.8 per 100 000 population [5]. Active case finding is crucial as is risk factor assessment and identification of drug resistant tuberculosis. The National Health Service now provides a wider TB care pathway to the public and aims to provide more effective health care to hard-to-reach groups such as prisoners, drug users and the homeless population [6]. Interferon gamma enzyme linked immunosorbent assay (Quantiferon test) and tuberculin skin test (TST) have been used in the detection of latent tuberculosis [3,7,8]. These are particularly useful in identifying tuberculosis among high risk populations such as migrants from high endemic areas.

Early diagnosis of active tuberculosis is vital for its treatment. It is usually achieved by identification of mycobacterium tuberculosis from body fluids such as sputum, bronchial lavage, synovial fluid from joints and tendons. Fine needle aspiration cytology of dactylitis is also a reliable and less invasive procedure to detect tuberculosis [9,10]. In children, lytic lesions seen in skeletal tuberculosis may resemble other sinister pathology such as leukaemia, neuroblastoma and Langerhan’s cell histiocytosis [11]. In these clinical circumstances, a tissue biopsy for Tuberculous PCR may prove to be of crucial diagnostic value [3, 12-14]. In recent years the role of radiology, in particular magnetic resonance imaging (MRI), has also been significant in early detection of suspicious cases and it is becoming increasingly popular.

Because of the current migration pattern, multi drug resistant tuberculosis is now well recognised not only in developing but also the developed countries. Patients with multi-drug resistant tuberculosis need expert care and should be referred to a specialist centre for optimal management.

This case shows that even in this day and age, extra pulmonary tuberculosis can be difficult to diagnose. It is therefore important to maintain a high index of suspicion in every person and not only in those who are immunocompromised. A multidisciplinary approach may assist in expediting a diagnosis and hence the treatment of extra pulmonary tuberculosis.
